# Bioavailability of a novel form of silicon supplement

**DOI:** 10.1038/s41598-018-35292-9

**Published:** 2018-11-19

**Authors:** D. V. Scholey, D. J. Belton, E. J. Burton, C. C. Perry

**Affiliations:** 1 School of Animal, Rural and Environmental Sciences, Nottingham Trent University, Brackenhurst Campus, Nottingham, NG25 0QF UK; 2 Interdisciplinary Biomedical Research Centre, School of Science and Technology, Nottingham Trent University, Clifton Lane, Nottingham, NG11 8NS UK

## Abstract

In this study, we assessed uptake and potential efficacy of a novel, pH neutral form of silicon supplement *in vitro* and using broiler chickens as a model species. *In vitro* bioavailability of this supplement was significantly higher than other commercial supplements tested, all of which claim available silica content. To confirm bioavailability of the new supplement *in vivo*, a broiler chick feeding trial reported blood uptake that was significantly higher than a Bamboo-derived silicon supplement. We assessed dose response of the novel supplement in a further study with increased dose related levels of silicon being detected in the blood and tibia. We found tibia and foot ash residue as a percentage of dry mass was higher with inclusion of the novel supplement in the diet, particularly in young birds and that this was followed by significant increase in tibia breaking strength. This novel supplement may therefore have applications in the improvement of bone integrity, with implications for the reduction of lameness in broilers. These results indicate the novel silica supplement is readily absorbed in chicks, and transported in the blood supply to sites such as the skeleton due to it being present in a non-condensed, monomeric form. There is potential for wider application of this silica supplement in other species where bone breakages are a problem, including high performance sport.

## Introduction

Silicon has a major function in developing structural integrity of biological organisms including higher plants^1^. In higher life forms, silicon is found in tissues including tendon, muscle, skin, vital organs and blood^2^. Early *in vivo* studies involving poultry^3^ and rats^4^ where controls were deprived of silicon, demonstrated that the element, in a bioavailable form, significantly affects growth and skeletal development. However, these early poultry studies were on a genetically different bird to today’s broilers. Silicon also appears to have a role in the early stages of bone regeneration following trauma^5^, but this is not yet fully understood.

The poultry industry is a rapidly growing sector where ongoing genetic improvements have increased bird growth rapidly over the last 50 years, but this has led to an increased incidence of skeletal disorders, resulting in lameness, mortality and more condemned carcasses at processing. Together these issues are major welfare and economic concerns for the poultry industry^6,7^. The potential for silicon supplementation to reduce the incidence of poultry lameness has been considered previously^8^, but difficulties with presenting the silicon in a form which is both bioavailable and non-toxic means these have shown limited progress to date^9^. In animal studies, synthetic silicon forms such as zeolites, alkoxy silanes, amorphous powders and highly caustic/acidic preparations have been adopted as a silicon source, with varying results^10–13^. The contrasting reported conclusions on the efficacy of silicon supplementation in reducing the incidence and severity of lameness may be due to differing physico-chemical composition and properties of different silicon supplements, which were not fully described in the published studies. Silicon (no source description) supplemented via the water available to broiler chickens has been shown to have no significant effect on bone breaking strength and bone density but did result in a change in the mineral profile of the bones with increases in phosphorus, zinc, copper, manganese and ash being observed^14^. The majority of naturally occurring silicon is present in highly stable minerals as silica and silicates. The stability of these minerals renders them highly resistant to dissolution into chemical forms that may be absorbed by the gastrointestinal tract and therefore are not bioavailable. However, weathering of rocks and soil minerals does produce, in low concentrations, a soluble, monomeric form of silica known as orthosilicic acid (Si(OH)_4_). Orthosilicic acid, the fundamental building block of biosilicas, is thought to be readily absorbed from the small intestine as its small molecular size and lack of charge allow it to pass easily through the mucosal layer of the gastro-intestinal tract^15^. Transit time for food in chickens can vary depending on intake rate but in general when fed ad libitum the retention time in the proventiculus and gizzard is about 2 hours (the crop is generally bypassed altogether) and the middle of the duodenum is reached after around another 0.5 hours. The ileum will be reached in around 5–6 hours^16^. By the time the ileum is reached the vast majority of digestion and absorption is complete, so the rate of release of orthosilicic acid from feed or supplements is critical. Orthosilicic acid is water soluble and weakly acidic (pK_a_ 9.8)^17,18^ but at neutral pH and concentrations greater than 2 mM, the monomer readily condenses to form insoluble polymers which eventually aggregate to form amorphous gel precipitates^17,18^. This precipitation and the increased molecular size and charge reduce its ability to pass through the mucus layer of the gastrointestinal tract and hence decreases its bioavailability^19^. Recent investigations in humans indicate a synthetic analogue of orthosilicic acid, monomethylsilanetriol, a monomeric, organosilicon molecule [Si(OH)_3_CH_3_] appears to be a non-toxic form of silicon that retains monomeric form in solution, but there is uncertainty over the *in vivo* biological capacity for cleavage of the Si-CH_3_ bond, which may limit bioconversion to the putative bioactive form, Si(OH)_3_OH^20^.

*In vitro* studies investigating collagen synthesis through culturing of human osteoblast-like cells show physiological concentrations of orthosilicic acid, the monomeric form of silica, increase collagen type 1 synthesis^21^, with smaller increases also seen in skin fibroblast cells. While these studies reinforce the idea that silicon may lead to improved skeletal integrity, this cannot be explored further until a form of silicon is produced that is bioavailable, non-toxic and affordable. The aim of this study was to determine the bioavailability and potential efficacy of a newly developed^22^, pH neutral form of silicon supplement using meat-type poultry as a model to observe skeletal effects.

## Results

Our studies encompassed both *in vitro* studies to compare the putative bioavailability of our pH neutral silicon supplement with a range of commercially available supplements and then comparative *in vivo* studies of the most effective supplement (novel silicon supplement MONO-Si) and a further commercially available supplement to assess both uptake and a first exploration of biological efficacy of the supplements. Note, these *in vivo* experiments cannot be directly compared with the early studies on poultry where a completely synthetic diet with total silicon removal from the diet was explored^3^ - this is no longer ethically possible. Our studies concentrate on looking at the effect of elemental supplementation on the birds.

**Figure 1:**
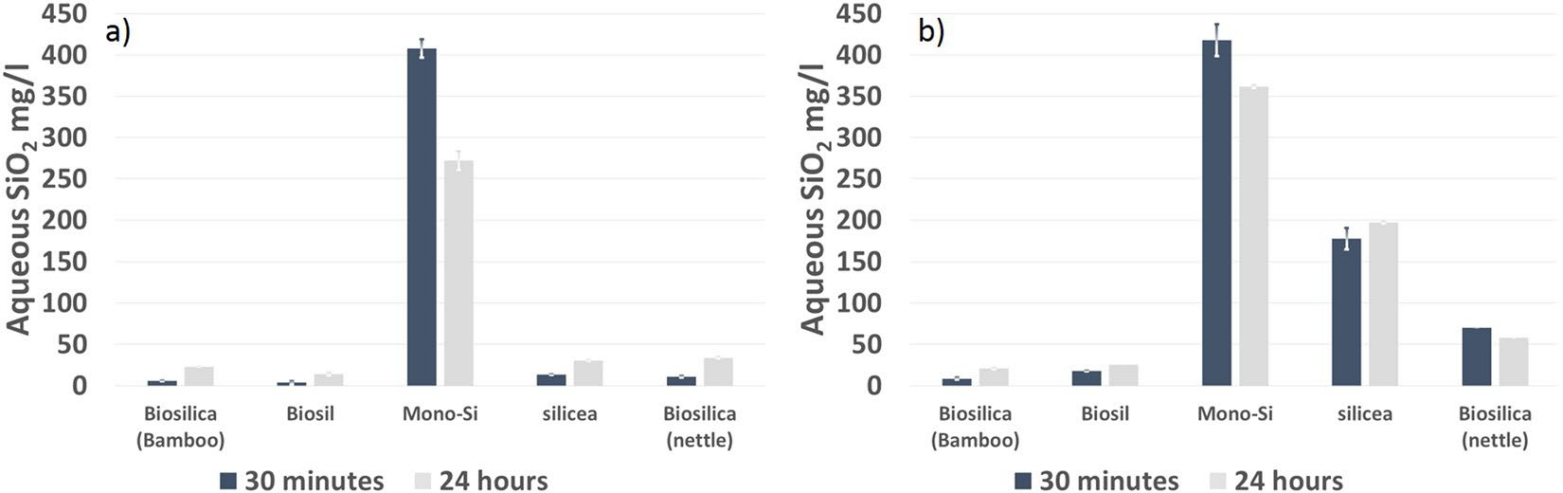
Bioavailability *in vitro* recovered from a novel silicon supplement MONO-Si (immediately after dispersion and after 24  hours in aqueous solution) versus commercially available silicon supplements (immediately after dispersion), all dosed at 500 mgl^−1^ as SiO_2_. (**a**) molybdenum blue analytical data; (**b**) ICP-OES analytical data.

For the *in vitro* investigations we used a proxy for bioavailability rather than previously reported digestive models^23^ due to the relative brevity of the feed transit process in chickens compared to humans (details below in materials and methods). Using this method we were able to measure significantly higher levels of bio-available silica from the novel silica formula providing 400 mgl^−1^ of bioavailable SiO_2_ (equivalent to 80% of the total silicon added) after sonication in water. Similar levels were not found for the four other commercial supplements tested (Fig. 1a) which only released 4–11 mgl^−1^ after the same treatment. Using both the molybdenum blue method (Fig. 1a) and ICP-OES (Fig. 1b) allowed us to distinguish between bioavailable and total suspended silica released by the various supplements and also the stability of the silicic acid solutions produced. A decrease over 24 hours was observed with the Mono-Si formulation as a result of supersaturated monomer solutions initially produced slowly condensing to silica and no longer being molybdenum blue active. The higher levels of silica species found for all of the samples tested using ICP-OES analysis is indicative of suspended particulate silica release especially by the silicea and nettle formulations. We would also say that the method we use to estimate bio-availability is more convenient for in the field *in vitro* testing of feedstocks.

The initial *in vivo* study compared the novel silica with a commercially available bamboo silica (Trial 1) and bird performance data for this is shown in Table 2, with no significant differences recorded across parameters between treatments. There were also no significant differences in bird feed intake or bodyweight gain between the novel silica (at any inclusion level) and the control in the dose response study carried out next (Trial 2, Table 3). Although there is a significant difference in feed conversion ratio in trial 2 this is unlikely to be supported by further studies, due to the low number of birds per pen at d42 resulting in high variation between pens.

In the first bird study, serum silicon content was significantly raised (*P* < 0.05) in birds fed diets supplemented with novel silica (5.3–6.2 mgl^−1^ Si in serum, Table 2) compared to birds fed diets supplemented with a Bamboo-Silica (2.4–2.6 mgl^−1^ Si) or those not fed any supplement (2.0–2.7 mgl^−1^ Si) in birds up to 5 weeks of age. There were no significant differences in serum silicon content between birds fed the control diet without supplement and those fed the diets with the commercial Bamboo derived silicon supplement at any time point. The monomeric silica also improved the bone breaking strength of tibias in birds at the point of slaughter, compared with the birds fed the control diet, but the strength of the bamboo silica fed birds was not significantly different from either group (Table 2).

**Figure 2:**
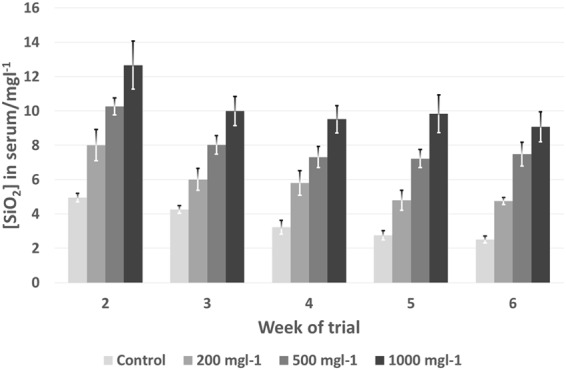
Serum silicon content of birds fed diets containing varying levels of novel silicon supplement (MONO-Si) during Trial 2 (pooled serum per pen, n = 6 pens per treatment).

In Trial 2 (dose response), Fig. 2 shows that serum concentration of silicon was highly significantly (*P* < 0.001) increased with silicon rate of inclusion in the diet and there was a similar enhancement over the control feed as seen in the previous trial. After week two, serum silicon concentration decreased across all treatments proportionately (even without silica augmentation), but the differences between treatments were still significant (*P* < 0.001 for all weeks).

At d14, both foot and tibia ash were significantly increased for the highest novel Si treatment compared with the control, and this positive effect was maintained in the 500 mg/l treatment diet until the end of the trial at d42 (Table 4). There were no significant differences for either foot or tibia ash percentage in Trial 2 in weeks 3 to 5. Table 4 also shows the measured silica content of the tibia ash, which is significantly increased at d21 and d28 for birds fed 1000 mg/kg novel silica compared with the control diet.

## Discussion

The potential for silicon in improving health and welfare of both humans and animals (in production or performance roles) has led to considerable interest in developing a stable, bioavailable form of silicon that could be used as a dietary supplement. A recent review article suggested that silicon might have a potentially positive effect on bone formation and density and support immunity in humans, but noted that commercially available silica sources were insoluble and therefore released very small amounts of orthosilicic acid^24^. The contrast between the abundance of naturally present silica in cereal-based animal feed and the bioavailable proportion is substantial: *in vitro* assessment of the basal (control diets) showed approximately 5% of the silica content (1 mg of a total 19 mgg^−1^) was found to be bioavailable. These solution concentrations of bioavailable species at ~20 mgl^−1^ are consistent with typical background levels found in natural water sources^19^. This confirmed the largely non-bioavailable nature of the silica contained in the control feed (largely in the form of phytolithic material from the grain content).

Several silicon supplements claiming high bioavailability are commercially available but reviews summarizing their bioavailability conclude them to be low or, at best, highly variable^19,25^. The *in vitro* analysis presented in Fig. 1 indicates that the novel form of silicon supplement^22^ investigated in this study was largely stabilized as the monomer or as a form which readily dissociates back to the monomer on dilution and is therefore bioavailable. By comparison, the commercial supplements were much less readily bioavailable with a considerable proportion of the silica released over time being of a particulate non-available nature and only providing levels of bio-availability similar to that found in local water supplies (typically 10–20 mgl^−1^ Si). This enhanced bioavailability was further emphasized by the significantly higher serum silicon content recorded in birds fed with MONO-Si in Trial 1 compared with those fed either the un-supplemented feed or feed augmented with the commercial supplement (Bamboo-Silica). This bioavailability is further evidenced by the linear increase in both serum and tibia silicon concentration seen with increasing supplementation level of MONO-Si as observed in the dose response study (Trial 2). These results confirm the feed concentration dependent transit of silicon species through the mucus layer of the gastrointestinal tract, and the slow release of bio-available silica observed with the biogenic silica could explain the observations of others who found Si level increases in the plasma in human studies of biogenic silica where we find none in the more rapid transit of chickens. We also noted that serum silicon concentration decreased across all treatments proportionately from week 2 onwards, and this general reduction in serum silicon level observed as trial 2 progressed may be indicative of the role of silicon in the early stages of bone deposition i.e. in the formation of the collagen matrix. A potential link between collagen production and silicon has been suggested previously^26^, particularly in relation to bone formation, but this requires further elucidation.

The effects of the novel silicon supplement presented in this study are of great interest as they are in contrast to earlier findings^13^, where the authors noted that, unless traditional conditions of extreme dietary silicon deficiency were imposed using synthetic diets^27^, no effect in broiler chickens was observed. These early studies were using birds which were very different genetically to modern broilers and therefore this may no longer be the case, in fact, the modern bird may be growing at such a rate that silicon is now deficient. It is well accepted in human medicine that the form of silicon provided as a supplement overwhelmingly influences its bioavailability^28^. The silicon form in MONO-Si is released into solution immediately as monosilicic acid, whereas many other Si sources present the silica in a condensed form (Fig. 1) which is apparently unavailable, resulting in low uptake in the bird as we observed in Trial 1.

The overall lack of impact of the silicon supplement on growth or feed intake ratio in Trial 1 or 2 (Tables 2 and 3) indicates that the palatability and overall performance of the diet was not affected by the inclusion of the silicon supplement. This is an important consideration in the development of novel feed supplements for any commercial feed industry, as any negative impact on performance renders the supplement uneconomical and conversely, any stimulation of increased growth rate may increase the physiological stress placed upon the birds. In Trial 1, the feed conversion ratio was numerically improved, but the lack of significant difference between treatments is likely due to the small number of birds per pen for performance measures.

Table 4 illustrates that percentage foot ash and tibia ash content were significantly higher in birds fed diets supplemented with 1 000 mg/kg MONO-Si compared to the control diet on d14, although this improvement did not continue to d42. This further suggests a role of silica in the early formation of bone, perhaps in the development of the collagen matrix, in young birds. If the effect of supplementation with bioavailable silicon is an improvement in construction of the collagen matrix, this may explain the improvement in bone strength in older birds seen in Table 2, where the MONO-Si improved strength of tibia bone in birds of slaughter age compared with control fed birds. Table 4 shows a modest but significant silica uptake in the tibia bone ash of around an additional 10% when the 1 000 mg/kg level MONO-Si doses were compared with the control. Silicon is known to be important in the formation of cross-linkages between collagen and proteoglycans in bone formation^29^ but defining its precise role in maintaining connective and skeletal tissue has proved elusive. It has been proposed^19,30^ that the biochemical role of silicon is in its involvement in DNA synthesis of osteoblast and extracellular matrix with structural function as a crosslink between procollagen linkage in collagen production and bone mineralization. *In vitro* studies have shown that human osteoblasts responded to silica gel to form nodules which eventually mineralized^31^ and dietary silicon intake was found to be significantly and positively correlated with cortical bone mineral density in men and pre-menopausal women^32^ but, once again, these studies have only postulated as to the mechanisms involving silicon. Recent research using genetic engineering created controlled silica mineralized silk films which were evaluated with human mesenchymal stem cells subjected to osteogenic differentiation demonstrated that the presence of the silica in the silk films influenced osteogenic gene expression, with the upregulation of alkaline phosphatase, bone sialoprotein, and collagen type 1 markers^33^. In addition, more recent studies of this type have shown that the induction of key markers of osteogenesis were correlated with the silica content of the materials^34^. These recorded effects provide possible mechanisms for the increased bone mineralization observed early in Trial 2.

Although positive *in vivo* effects of silicon supplementation on bone formation and connective tissue metabolism were reported in earlier studies^27^, other studies have reported no effects^9^. It has also been reported that silicon supplementation of tetraethylorthosilicate in rats and turkeys resulted in lower bone size and strength parameters such as those measured by maximal load and elasticity of the femur and tibia bone but these conclusions were based on numerical rather than statistically significant differences^35^. These contradictory data highlight the importance of providing a form of silicon in the diet that not only remains bioavailable but will not undergo negative interactions with other minerals. The lack of apparent lameness observed in any birds combined with the increased tibia silicon content observed in Trial 2 with MONO-Si supplementation, suggests that it is unlikely that an adverse interaction between the current supplement and other minerals involved in bone development is occurring. Larger scale trials or intentional induction of lameness in future studies will be necessary to determine if the novel silicon supplement alleviates lameness in poultry. This type of study would also allow further investigation into the potential mechanisms behind the role of silicon in bone and tissue development and repair that have been previously outlined in rabbits^5^, poultry^3^ and in humans^32^.

The establishment of this silicon supplement as bioavailable to broiler chicks from hatch and throughout the growth phase to slaughter weight, warrants future studies to examine both the efficacy of the supplement in alleviating lameness in poultry and also the mode of action for silicon’s involvement in maintaining tissue integrity. Oral ingestion toxicity levels have not been established for humans so establishment of a safe upper level is based on a rat and mice toxicity study that concluded that the chronic toxicity of silicon is low^36^. The Expert group on Vitamins and Minerals (EVM)^37^ set a Safe Upper Level for daily consumption of silicon at 700 mg silicon/day for adults over a lifetime (equivalent to 12 mg silicon/kg body weight/day for a 60 kg adult). Preliminary data indicating safe upper limits for oral ingestion of silicon in poultry have been determined using turkeys^35^ but ensuring proposed supplement levels are safe and comply with required labelling standards^38^ will still need to be considered before marketing this product for use in poultry or humans. Whilst this communication has focused on the potential use of bioavailable silicon in poultry, there are many other spheres of animal production, human health and high performance sport where a bioavailable form of silicon may also be highly beneficial in maintaining and potentially repairing skeletal integrity.

The novel monomeric form of silica developed by the authors is significantly more bioavailable *in vitro* compared with a range of commercially available formats. Bird trials using broiler chicks have shown that this form of silica can be taken up by the bird into bone and serum and improves bone quality and strength, thereby helping alleviate the lameness commonly seen in the broiler industry. This preliminary study has future implications for other fields where improved bone integrity would be beneficial.

## Material and Methods

### Experimental design

Five forms of silicon-based health supplement were initially screened using an *in vitro* assessment of bioavailability, before undertaking feeding trials in broiler chicks. The first broiler chick trial compared uptake into serum of a novel form of silicon supplement with a form currently commercially available while the second broiler chick trial examined the effect of incremental dosage of the supplement which had been identified in trial 1 as having the highest serum uptake.

### *In vitro* bioavailability of silicon supplements

For the *in vitro* experiment described in this contribution, bioavailable silicon is defined as monomeric silicic acid and silicate species, which rapidly dissociate to the monomer, as quantified by the colorimetric molybdenum blue method^39^. Soluble/dispersible silicon is defined as monomeric, oligomeric and polymeric silicates which dissolve or suspend readily to give stable colloids in aqueous media, only a proportion of which may be bioavailable, and are quantified by inductively coupled plasma optical emission spectrophotometry (ICP-OES). Total silicon is defined as soluble/dispersible silicon plus larger silicate particles which do not suspend in aqueous media but require initial dissolution in alkaline hydroxide solutions (e.g. sodium or potassium) in order to reduce the condensed silica back to monomeric form before quantification by either the colorimetric molybdenum blue assay or ICP-OES.

*In vitro* bioavailability was compared for five forms of silicon supplement: a mineral-derived colloidal silica (Hubner Silicea^TM^, Anton Hubner, GmBH and Co, Germany), a choline-stabilised nanoparticulate colloidal silicic acid source (Biosil, Biominerals, N.V., Belgium), silica of biogenic origin from nettles (Enzymatic Therapy, INC. USA) silica of biogenic origin from bamboo (Nature’s Best, UK) and a newly developed pH-buffered, monomeric source of silicic acid^22^ (‘MONO-Si’). All supplements were dosed at 500 mgl^−1^ (as SiO_2_) in water, based on their declared silica content for assay. Bioavailable silicon content was analysed by a molybdenum based colorimetric assay^39^ developed from the method originally reported by R.K. Iler^17^ (detail provided in SI1).

### *In vivo* bioavailability of silicon supplements

Two broiler chick assays, both using male Ross 308 strain chicks from PD Hook (Oxford, UK), were undertaken to evaluate the newly developed silicon supplement (MONO-Si) *in vivo*. Institutional and UK national NC3R ARRIVE guidelines for the care, use and reporting of animals in research^40^ were followed and all experimental procedures involving animals were approved by the Nottingham Trent University College of Science Animal Welfare and Ethical Review Committee. Neither study was deemed to require licensing under the UK Animals (Scientific Procedures) Act (1986) Amendment Regulations (2012) by the UK regional Home Office Inspector. Further details relating to bird husbandry are provided in SI2.

### Dietary treatments

For bird trial 1 (comparison of serum silicon levels of birds fed diets containing differing silicon supplements), the silicon supplements were fed as part of a nutritionally balanced, wheat-soya-rapeseed based mash diet made in house as described below. The MONO-Si silicon supplement was suspended in soya oil before adding to the basal starter/finisher feed and the Bamboo-Si silicon supplement was added as a powder to the manufactured feed. A commercially formulated starter basal diet was was used from days 0 to 21 and a finisher diet from days 22 to 35 (Table 1). Birds were fed either the basal diet with no supplemental silicon (Control Diet) or diets containing either MONO-Si or Bamboo silicon supplement included at 1 000 mgkg^−1^ as SiO_2_ in the final feed mixes (MONO-Si diet and Bamboo-Si diets respectively). The total silicon content of the control diet was 4.7 g/kg in the starter diet and 6.4 g/kg in the finisher diet (determined by ICP-OES analysis of a sodium hydroxide digest). Dietary treatments were allocated to 27 pens with 9 pens (experimental units) fed each treatment. Birds were housed in groups of 7 birds to d14, then 4 birds to d21 and 2 birds to d35. Birds (three per pen at week 2; two per pen for weeks 3 and 5) from each treatment were euthanized to obtain samples for analysis.

**Table 1:** Material composition of broiler starter and finisher diets for Trials 1 and 2.

Raw material	Rate of dietary inclusion, g/kg as fed			
	Trial 1 Starter diet	Trial 1 Finisher diet	Trial 2 Starter diet	Trial 2 Finisher diet
Ground wheat	632	717	637	682
Full fat rapeseed meal	40	40	—	—
Extruded high protein soya	260	183	250	175
Extruded full fat soya	—	—	25	50
Soya oil	36	35	40	40
Salt	3.0	3.0	2.5	2.5
Sodium bicarobonate	1.0	1.0	1.5	1.5
DL methionine	2.8	1.5	2.5	3.0
Lysine HCl	2.6	2.1	3.0	3.9
Threonine	0.7	0.4		
Limestone	9.1	8.7	15	13
Dicalcium phosphate	8.7	3.7	—	—
Monocalcium phosphate	—	—	15	13
Vitamin mineral premix	5.0	5.0	3.5	3.5
Choline chloride	—	—	0.5	0.5

**Table 2:** Mean feed intake (FI), bodyweight gain (BWG) and feed conversion ratio (FCR) of broiler chicks fed either a novel monomeric silicic acid (MONO-Si) or Biogenic silicon derived from bamboo (Bamboo-Si) (n = 9 per treatment) for 35 days during Trial 1, including serum silica of birds at day 14 and 35 and bone strength of tibia bones at d35 (SEM refers to standard error of the mean). The use of superscipt letters denotes statistical differences within a row at P > 0.05.

	Control	MONO-Si	Bamboo-Si	SEM	p value
D0-35 FI/bird (g)	3140	3236	3317	88.8	0.444
D0-35 BWG/bird (g)	1975	2055	1951	48.4	0.377
FCR d0-35	1.60	1.59	1.70	0.041	0.12
Serum Si d14 (mg/l)	2.67^b^	6.19^a^	2.64^b^	0.197	<0.001
Serum Si d35 (mg/l)	2.04^b^	5.31^a^	2.44^b^	0.143	<0.001
Bone strength d35 (N)	344^b^	420^a^	379^ab^	14.5	0.049

**Table 3:** Mean daily feed intake (FI), d42 body weight gain (BWG), and Feed conversion ratio (FCR) of broiler birds fed graded levels of silicon supplement MONO-Si; (n = 6 per treatment) for 42 days during Trial 2 1 (SEM refers to standard error of the mean).

	Silica content of diet mg/kg					
	0	250	500	1000	SEM^1^	p value
D0-42 FI (g/bird/d)	78	81	79	81	1.7	0.574
D0-42 BWG/bird (g)	2630	2617	2757	2514	93.8	0.434
FCR d0-42	1.51	1.56	1.49	1.59	0.025	0.023

**Table 4:** Foot and tibia ash % content, and tibia silicon contents (mg/kg tibia ash weight) of broiler chicks fed diets containing varying levels of novel silicon supplement (MONO-Si) up to day 42 during Trial 2 (n = 6 pens per treatment).

	Dietary Silica (MONO-Si) content mg/kg				SEM^2^	p value
	0	250	500	1000		
D14 Foot ash (%)	14.7^c1^	16.3^ab^	15.6^bc^	16.9^a^	0.31	<0.001
D14 Tibia ash (%)	46.1^b^	48.9^ab^	50.4^ab^	51.9^a^	1.14	0.035
D42 Foot ash (%)	15.1	14.7	15.6	15.2	0.37	0.368
D42 Tibia ash (%)	44.2^ab^	45.0^ab^	46.2^a^	43.5^b^	0.60	0.031
D21 Tibia Si (mg/kg)	20.0^b^	21.0^ab^	22.4^a^	22.4^a^	0.47	0.003
D28 Tibia Si (mg/kg)	18.1^b^	19.0^ab^	20.0^ab^	20.1^a^	0.45	0.02
D35 Tibia Si (mg/kg)	21.9	22.7	23.6	23.9	0.71	0.235
D42 Tibia Si (mg/kg)	18.4	18.6	20.3	20.0	0.69	0.153

Differing superscript letters within a row denote means are significantly different at P < 0.05. SEM refers to standard error of mean.

For bird trial 2 (Investigation into the effects of increasing levels of silicon supplement on silicon content in serum and tibia and foot ash content from hatch to slaughter age) birds were fed a commercially produced starter basal diet (Target Feeds Ltd, Shropshire, UK) from days 0 to 21 and a finisher diet from days 22 to 42 (Table 1) with treatments as described below. The MONO-Si silicon supplement was suspended in soya oil and added to a nutritionally balanced, wheat-soya based mash diet at 0, 200, 500 and 1 000 mgkg^−1^ as SiO_2_. Each basal diet had 20 g/kg of the soya oil omitted at manufacture and subsequently this was used to suspend the appropriate volume of silicon supplement, before adding the oil back to the basal diet to create each experimental diet. The silicon content of the control diet was 5.3 g/kg in the starter and 6.1 g/kg finisher diet (determined as per trial 1). Treatments were allocated to six pairs of pens with each pair of pens counting as one experimental unit. The birds were housed in pens of six for 2 weeks and then in decreasing numbers during the study. Each week birds in each experimental unit were weighed. Feed intake was measured for weeks 0–3 and 3–6 for each experimental unit. Feed intake was calculated to give grammes of feed eaten per bird per day and feed conversion was determined. Birds (two per pen at 2 weeks of age and thereafter 1 bird per pen weekly) from each treatment were euthanized to obtain samples for analysis.

### Measurement of serum silicon content

In both bird trials, birds were euthanized using cervical dislocation and post mortem blood samples were collected. Blood was removed using a syringe and left to clot in a 15 ml centrifuge tube at room temperature. Tubes were then centrifuged at 1500 g for 10 minutes and the serum frozen at −20 °C until required for analysis. Serum samples were analysed by ICP-OES as described in SI1.

### Measurement of bone mineral content

Tibia ash was determined by manual removal of flesh from the bones, before drying to constant weight, and ashing for 13 h at 650 °C. Foot ash was determined as for tibia ash, but without any flesh removal prior to drying. Percentage bone ash was calculated as ash weight as a proportion of dry bone/foot weight. Silica content of the bones was analyzed by ICP-OES analysis as described in SI3.

### Measurement of bone strength

Tibias were removed between the tibial-tarsal joint and the tibial-femoral joint. using a TA. XT plus texture analyzer (Stable Microsystems, Guildford, UK) set up with a 50 kg load cell and 3 point-bend fixture^41^.

### Data analysis

Data were analysed using SPSS version 12 to determine One way Analysis of Variance using a two tailed analysis with rate of silicon inclusion considered as a treatment factor. Treatment means were separated using Bonferroni’s post hoc test and statistical significance was declared at *P* < 0.05.

## Electronic supplementary material

Supplementary Information

## Data Availability

Data will be made available upon reasonable request.
