# Supplementary material for: Bioavailability of a novel form of silicon supplement
Source: Sci Rep. 2018 Nov 19;8:17022. doi: 10.1038/s41598-018-35292-9 (PMC6242837; doi:10.1038/s41598-018-35292-9)
Supplement: Supplementary file 1 — Supplementary Information [file 41598_2018_35292_MOESM1_ESM.docx]

**Bioavailability of a novel form of silicon supplement.**

D. V. Scholey^1*^, D. J. Belton^2^, E. J. Burton^1^ and C. C. Perry^2*^

^1^School of Animal, Rural and Environmental Sciences, Nottingham Trent University, Brackenhurst Campus, Nottingham, NG25 0QF, UK.

[*Dawn.scholey@ntu.ac.uk*](mailto:Dawn.scholey@ntu.ac.uk)*; Emily.burton@ntu.ac.uk*

^2^Interdisciplinary Biomedical Research Centre, School of Science and Technology, Nottingham Trent University, Clifton Lane, Nottingham, NG11 8NS, UK [*David.belton2@ntu.ac.uk*](mailto:David.belton2@ntu.ac.uk)*; Carole.perry@ntu.ac.uk*

**Supplementary information 1 (SI1)**

Method for measuring Silica bioavailability: Molybdenum blue method:

Briefly, solutions of the Silicon supplement (equivalent to 500 mgl^-1^ as SiO_2_) were prepared by 30 minutes sonication followed by sedimentation by centrifugation at 2900g for 5 minutes. Aliquots of the supernatant were added to diluted molybdic acid reagent (ammonium molybdate, 2% in dilute hydrochloric acid (0.6M); 1.5 cm^3^ diluted with 15 cm^3^ distilled and deionised water) and the silicomolybdic acid complex allowed to develop for 15 minutes. 8cm^3^ of reducing reagent containing oxalic acid to quench phosphorous interference (2% oxalic acid, 0.67% 4 methyl amino phenol and 0.4% sodium sulphite in 1.9M sulphuric acid) was then added and the blue silicomolybdous acid complex allowed to develop. The absorbance of the samples was measured at 810nm, a minimum of 2 hours and maximum of 48 hours after addition of the reducing reagent (complete reduction requires 2 hours and the complex solution is stable for at least 48 hours), and the results compared against 1 - 10 mgl^-1^ SiO_2_ standards. Bioavailability recovered from the silicon supplements was then calculated as bioavailability mgl^-1^ / as SiO_2_.

Method for measuring total silicon concentrations (ICP-OES)

For ICP-OES analysis, samples (50 mg 100ml^-1^ as SiO_2_) were dispersed by 30 minute ultrasonication in 18 ohm water. Immediately 10ml aliquots were taken and centrifuged at 4000rpm for 5 minutes and then 100µl of the supernatant diluted to 10.0ml with 18ohm water. Analysis was by ICP-OES (Perkin Elmer ICP DV2100) using the 251.611nm emission line for silicon and measuring against a set of 0.1 – 20ppm silica standards. The unused centrifugate was redispersed and returned to the bulk dispersion and stored for 24 hours with occasional mixing. The centrifugation and analysis steps were then repeated as before.

Serum samples were analysed as follows: samples were diluted x 10 with 18ohm H_2_O and analysed by ICP-OES (Perkin Elmer ICP DV2100) using the 251.611nm Si emission line and compared with 0 – 10 ppm SiO_2_ standards.

**Supplementary information 2 (SI2)**

In both bird trials, birds were housed from day old in 80 x 80cm floor pens in an environmentally controlled room with wood shavings as a litter substrate. Feed was provided via troughs attached to the front of the pen and water by bell drinkers. Feed and water were provided *ad libitum*. The silicon dosage used did not exceed the maximum advisable intake of silicon for poultry, based on established data from experts on vitamins and minerals^34^. Pen temperature was set at 33°C at day old decreasing to 21°C by 21 days of age and remaining at 21°C until the end of the study. The lighting was set at 23 hours on with 1 hour dark.

**Supplementary information 3 (SI3)**

To analyse Si content of the tibia bones, approximately 0.5 g of bone ash was accurately weighed, dissolved in and diluted to 10.0 cm^3^ with *aqua regia* (70% HNO_3_ /35% HCl in a 1:3 ratio). The samples were left to fully digest and degas to a clear solution at room temperature overnight. 200µl digest was diluted to 10.0ml using 18ohm water and the silicon content determined as SiO_2_ by ICP-OES analysis as described above in SI1.
